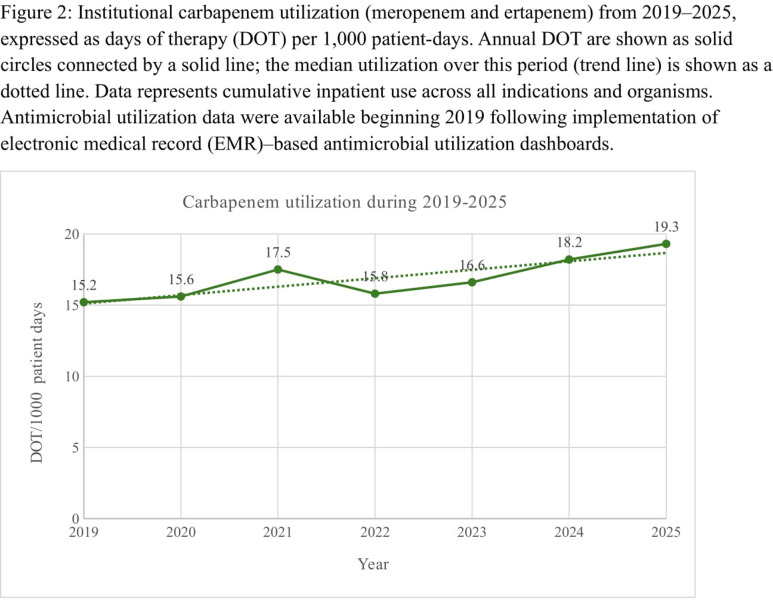# 62 National Data Indicates Increases in the Burden of Clostridioides difficile infections in the United States Following COVID-19 Pandemic

**DOI:** 10.1017/ash.2026.10493

**Published:** 2026-06-23

**Authors:** Ayesha Samreen, Omar Abu Saleh, Lynn Estes

**Affiliations:** 1 Mayo Clinic, Infectious diseases; 2 Mayo Clinic

## Abstract

**Background:** Ceftriaxone and cefepime resistance among Enterobacterales continues to increase globally, partly due to the rising prevalence of extended-spectrum β-lactamase (ESBL) producing organisms. Surveillance of both resistance patterns and antimicrobial utilization is essential to inform empiric prescribing practices and stewardship strategies. Since carbapenems are frequently used to treat cephalosporin-nonsusceptible Enterobacterales infections, we evaluated trends in ceftriaxone and cefepime susceptibility among Escherichia coli and Klebsiella pneumoniae from 2012–2024 and institutional carbapenem utilization during overlapping years (2019–2025), following outcomes from the MERINO trial for management of ceftriaxone-resistant Enterobacterales infections. **Methods:** We conducted a retrospective review of annual cumulative inpatient antibiogram data for E. coli and K. pneumonia isolates for 2012-2024. Percent susceptibility to ceftriaxone and cefepime was assessed using Clinical and Laboratory Standards Institute (CLSI) breakpoints among a median of 1,532 E. coli isolates/year and 561 K. pneumoniae isolates/year. (Table 1). Institutional carbapenem utilization (combined meropenem and ertapenem use) was measured as days of therapy (DOT) per 1000 patient-days during 2019–2025 (not specifically representing treatment of E. coli or K. pneumoniae), following implementation of electronic medical record (EMR)–based antimicrobial utilization dashboards. Temporal trends were visualized using line plots. Pearson correlation coefficients (r) were calculated to assess percent susceptibility trends and carbapenem utilization during 2019–2024. Analyses are descriptive; no causal inference was made. **Result:** During 2012–2024, ceftriaxone susceptibility declined from 89% to 83% among E. coli and from 93% to 82% for K. pneumoniae, while cefepime susceptibility declined from 95% to 88% and 97% to 85% respectively (Figure 1). During the overlapping years 2019–2024, Pearson correlation showed modest downward susceptibility trends for ceftriaxone (E. coli r = −0.900, p = 0.014; K. pneumoniae r = −0.840, p = 0.036) and cefepime (E. coli r = −0.780, p = 0.067; K. pneumoniae r = −0.850, p = 0.033). Institutional carbapenem use increased from 15.2 to 19.3 DOT per 1,000 patient days between 2019 and 2025 (r = 0.79, p = 0.035) (Figure 2). Upon review, carbapenem use was substantially higher in hematology-oncology services (23.7–25.9 DOT/1000 patient-days, during 2022–2025) compared with overall institutional use (15.2–19.3 DOT/1000 patient-days). **Conclusion:** Ceftriaxone and cefepime susceptibility among Enterobacterales declined over the past decade, coinciding with a modest increase in institutional carbapenem use without a corresponding increase in carbapenem resistance during 2019-2024. Local susceptibility patterns and antimicrobial utilization data provide critical context for stewardship prioritization, especially in high-risk hematology-oncology populations.

**Figure:**
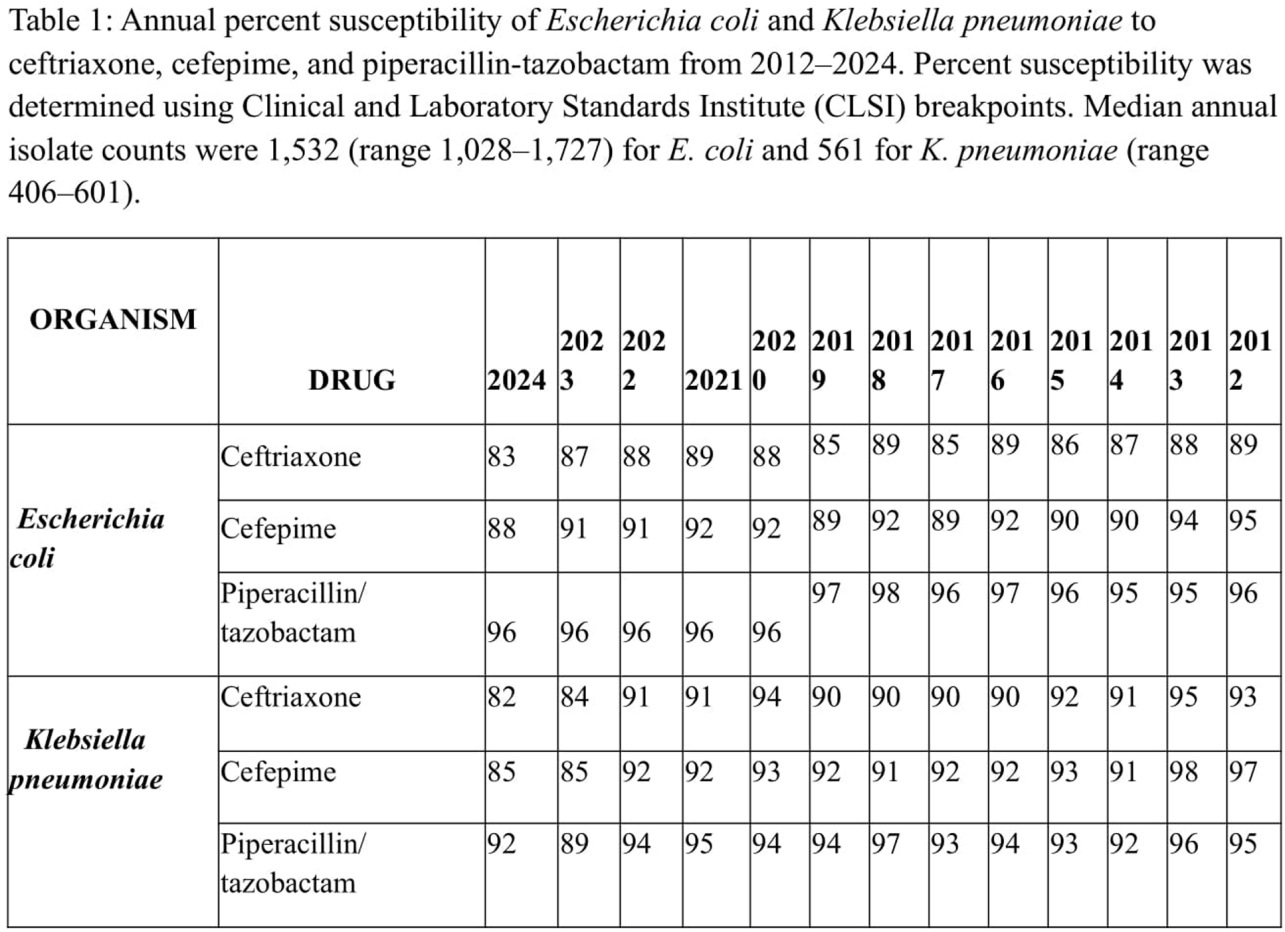

**Figure:**
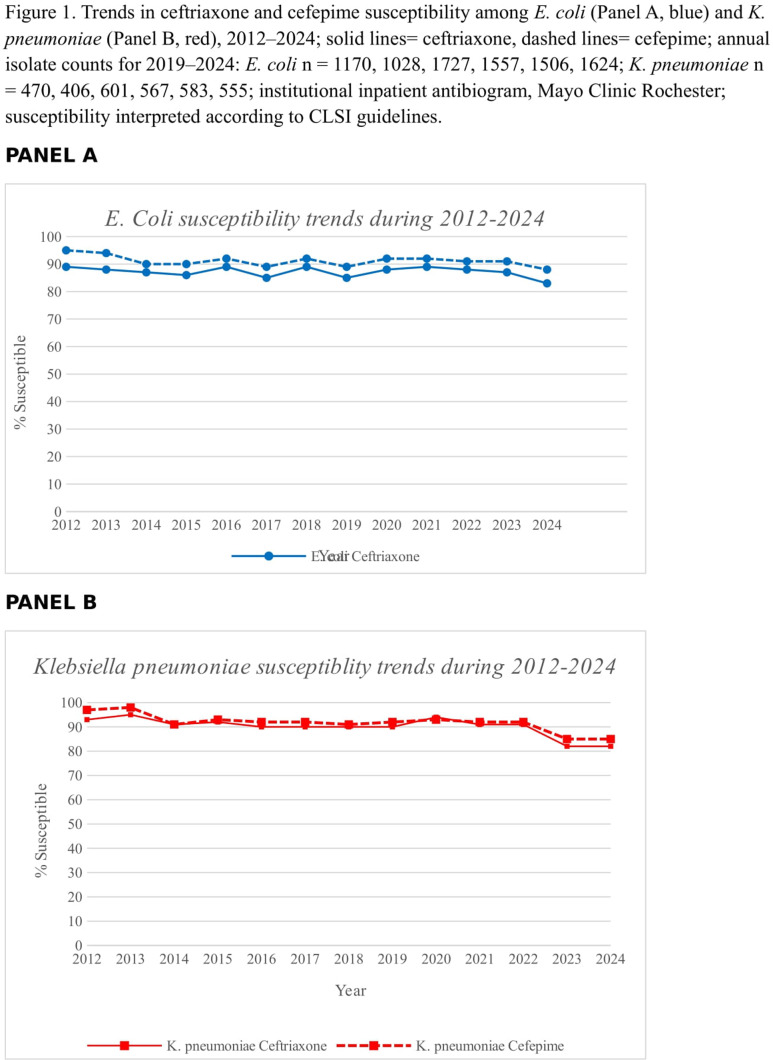

**Figure:**